# Comparative Ecology of *Hyalomma lusitanicum* and *Hyalomma marginatum* Koch, 1844 (Acarina: Ixodidae)

**DOI:** 10.3390/insects11050303

**Published:** 2020-05-13

**Authors:** Félix Valcárcel, Julia González, Marta G. González, María Sánchez, José María Tercero, Latifa Elhachimi, Juan D. Carbonell, A. Sonia Olmeda

**Affiliations:** 1 Grupo de Parasitología Animal, Animalario del Departamento de Reproducción Animal, INIA, 28040 Madrid, Spain; marta.glez.degano@gmail.com (M.G.G.); maria2985@hotmail.com (M.S.); 2 Villamagna S.A., Finca ‘‘La Garganta’’, 14440 Villanueva de Córdoba, Spain; julsglezglez@gmail.com (J.G.); jmtercero@fincalagarganta.com (J.M.T.); 3 Center for Vector Biology, Department of Entomology, Rutgers University, New Brunswick, NJ 08901, USA; 4 Département de Parasitologie et Santé Publique, Institut Agronomique et Vétérinaire Hassan II, Rabat-Instituts B.P. 6202, Morocco; latifa.elhachimi@gmail.com; 5 Departamento de Sanidad Animal, Facultad de Veterinaria, UCM, 28040 Madrid, Spain; juan.carbonell.bonelo@gmail.com (J.D.C.); angeles@ucm.es (A.S.O.)

**Keywords:** *Hyalomma lusitanicum*, *Hyalomma marginatum*, ecology

## Abstract

The genus *Hyalomma* belongs to the Ixodidae family and includes many tick species. Most species in this genus are African species, but two of them, *Hyalomma lusitanicum* and *Hyalomma marginatum,* are also found in Europe and, owing to their morphological similarity, it is very difficult to tell them apart. This is a major concern because their phenology and vector capacities are quite different. Moreover, they share many habitats and both are currently spreading to new areas, probably due to climate change and animal/human movements. In this study, we review the described ecology of the two species and provide further interesting data on *H. lusitanicum* based on the authors’ experience, which could be useful in assessing the risk they pose to humans and animals.

## 1. Introduction

Ticks are blood-sucking ectoparasites, present throughout the world, and are very important vectors of many pathogens. The Ixodidae family (hard ticks) is comprised of approximately 896 species [1], many of which are morphologically quite similar. Some species, only distinguishable by experts, have different ecological patterns and vectorial capacities. Attention has recently turned to two species of the *Hyalomma* genus, *Hyalomma lusitanicum* and *Hyalomma marginatum* [2,3,4,5], which have recently begun to spread to areas of Europe outside of their natural habitat [6,7,8]. They are increasingly found in many parts of Northern Europe [9,10,11,12,13,14], probably because of climate change as well as human and animal movements [15,16]. This is significant because both species are known to be vectors of diseases to human and/or animals; *H. marginatum* is the main known vector of the Crimean-Congo Hemorrhagic Fever virus (CCHF-v) [11] and *H. lusitanicum* transmits *Theileria annulata*, agent of Mediterranean theileriosis [17], and more recent data confirm its role in the transmission of *Coxiella burnetii*, the agent of Q-fever [18,19], among other pathogens (Table 1).

As mentioned above, *H. lusitanicum* and *H. marginatum* have similar morphology and are difficult to identify owing to their close taxonomic relationship, which is why some recent studies have focused on morphological descriptions [29,30]. However, once identified, the real risk of transmission of tick-borne diseases (TBD) to humans and animals must be determined. TBD prevalence in each area mostly depends on tick behavior and host availability, but this is not an easy task as many aspects need to be evaluated under subjective criteria that are difficult to measure. Information regarding *H. lusitanicum* ecology is limited to a few recent studies in the Iberian Peninsula [31,32,33,34,35,36], while for *H. marginatum*, most reports are focused on morphological descriptions but offer scant data on its presence on certain hosts or its role as a vector of CCHF-v [11,20,30]. Moreover, there is a lack of information on potential health risks in non-endemic areas where they are increasingly being found.

The aim of this study is to summarize their ecological and behavioral characteristics and to try to redefine those which could be confused in the literature by providing new data on the ecological differences between the two species. This information could be useful in developing control strategies when needed.

## 2. Materials and Methods

The main ecological aspects of two tick species of the *Hyalomma* genus, *Hyalomma lusitanicum* and *Hyalomma marginatum,* have been reviewed. Entering the key word *Hyalomma lusitanicum* into a search of the electronic bibliographic database Pubmed (https://www.ncbi.nlm.nih.gov/pubmed on 18 February 2020) produced only 63 hits spanning from 1980 to 2020, while the same search for *Hyalomma marginatum* produced 433 hits from 1950 to 2020. In this study we mainly considered articles that specifically described one or more aspects of the life cycle, host affinity, questing/dragging strategies, host infestation, sex ratio or these species’ relations to climate, habitat and geographical distribution. We also considered several reviews of the genus *Hyalomma* that focused on morphological descriptions but that also offered some biological information. Other articles which simply reported on their presence on a particular host or in a certain area were considered when relevant. Publications on ecological aspects comparing the two species were complemented with personal observations based on our 14 years of experience studying and controlling *Hyalomma* ticks in a meso-Mediterranean area. Some results of our observations have previously been reported [19,29,31,32,33,34,35,36,37,38,39,40,41,42] but others remained unpublished until now. These unpublished data are indicated in the text and tables as personal observations. Several short videos are also available as Supplementary files.

## 3. Results and Discussion

*Hyalomma* ticks are usually bi- or tri-phasic (two or three-hosts), depending on whether or not larvae molt. *Hyalomma lusitanicum* is a three-host tick where each stage feeds on a different host and all tick stages, once engorged, detach to molt on the ground. *Hyalomma marginatum* is a two-host tick (although under certain conditions, such as artificial infestation, could be a three-host tick [43], so the engorged larvae usually remain on the host to molt and feed as nymphs (Table 2). Therefore, the main difference in the life cycle of these two species is the place where engorged larva molt and the ensuing nymphs feed, i.e., the same host as larva in *H. marginatum* and different hosts in the case of *H. lusitanicum*. The rest of the life cycle is similar: engorged nymphs molt on the ground, adults feed on another host, and gravid females which reach its optimal weight drop off and lay eggs on the ground [44]. The males of both species remain on the host where they can mate with many females [11,35].

**Figure 1:**
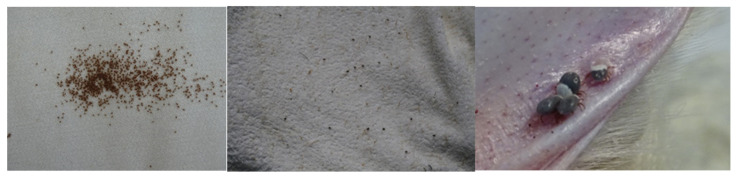
*Hyalomma* larvae nest (left) may be occasionally collected by dragging in open field. Host seeking *Hyalomma lusitanicum* nymphs (center) may also be collected by dragging, but not very often because they are usually endophilic. However *Hyalomma marginatum* is diphasic and larvae molt on the host (right) in consequence unfed nymphs cannot be found by dragging.

Immature stages of both species are similarly classified as endophilic/exophilic, remaining most of the time in the nest of hosts [30], but it is important to highlight a few aspects. Gravid females lay their eggs in the soil and by dragging it is possible to find groups of thousands of newborn larvae searching for hosts. Due to its biphasic cycle, it is not possible to find host-searching nymphs of *H. margitanum* in open environments while, although unusual, is possible to find *H. lusitanicum* nymphs by dragging [30] (Figure 1). This can be observed in areas where there is a reduced population of its main host, wild rabbit [53,54], or when the local tick population is very high. Adults of both species are exophilic and spend most of their life waiting for a host in the field, but other authors report that all stages of *H. marginatum* may be endophilic or exophilic [45], probably because of the difficulty in finding adults by dragging, as will be discussed later in this paper.

### 3.1. Host Affinity

*Hyalomma* spp. adults usually feed on large animals while preadults feed on small ones. According to Apanaskevich [55], most *Hyalomma* species (*H. marginatum*, *H. schulzei, H. excavatum, H. asiaticum, H. franchinii, H. impeltatum, H. impressum*, *H. lusitanicum*, *H. nitidum, H. truncatum and H. albiparmatum*) should be considered ditropic since immature stages only prey upon small mammals and birds while adults parasitize large mammals. However, some tick species (*H. dromedarii, H. anatolicum* and *H. scupense*) should be classified as monotropic insofar as all stages parasitize large mammals [30]. There are many hosts on which *H. lusitanicum* and *H. marginatum* are usually ditropic, but there some differential characteristics regarding host affinity or location on the host (Table 2).

Due to different host affinities and the number of hosts and their respective life cycles, several different host infection patterns could emerge depending on the animal species, and tick stage and species. Adult stages of both species feed on many domestic and wild hosts but it would appear that *H. lusitanicum* is more frequently found on wild animals whilst *H. marginatum* more frequently infests domestic animals as has been reported in Africa [30]. A common characteristic of both species studied is that their prevalence is increasing. For example, at the end of the 1990s in the Iberian Peninsula, *H. lusitanicum* adults infested 46 to 75% of red deer [56], but today, infestation affects nearly 100% of red deer [35]. In 2004, *H. marginatum* accounted for less than 1% of the *Hyalomma* ticks infesting cattle in Turkey [57]. However, one year later, 85% of the ticks collected from this ruminant were *H. marginatum* [58]. Furthermore, monthly distribution is also expanding, as well as its geographical distribution (see geographical section).

As mentioned, *H. lusitanicum* is usually classified as a ditropic tick [21,32,59,60] given that immature stages mainly feed on wild rabbit and adults on ungulates but immature stages are also able to feed on the same types of hosts as adults and this is not uncommon [35]. This classification must be considered taking preferred hosts into account at each stage, but they could occasionally feed on other animals or secondary hosts (Table 2). Hence, despite the descriptions of Walker et al. [30], among others, *H. lusitanicum* should be re-classified as a telotropic species. These findings are consistent with reports on the presence of immature stages feeding on wild birds and red deer [61,62,63], which may help resolve the doubt expressed by Apanaskevich et al. [64] regarding the need to confirm the studies conducted by García Fernández and Hueli and Perez-Eid and Cabrita [46,47], who reported the presence of immature stages of *H. lusitanicum* on cattle and domestic birds.

While *H. lusitanicum* tends to attach on ears in rabbits and on the belly and perineal area of ungulates, they are widespread over the entire host [35]. *H. marginatum* could be monophasic (using ungulates as the only host for all stages) [16] but, as a general rule, adults prefer to feed on ungulates, while immature stages feed on leporids (rabbits or hares) and several passerine bird species, but not on rodents [58] (Table 2). As for feeding sites on ruminants, *H. marginatum* usually congregates around the hind quarters, especially the udder, scrotum, inguinal area, and perineum; on birds and small-medium-sized wild mammals, ticks are also commonly found around the head, in particular in and around the ears; on hares, larvae attach to ear edges and nymphs attach to the face, neck, and around the eyes [11,65]. In contrast, *H. lusitanicum* does not usually congregate (personal observations). The reason for that difference is not clear.

**Figure 2:**
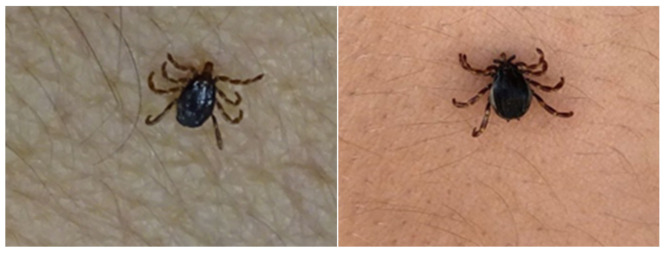
*Hyalomma marginatum* attempting to attach immediately opening its palps and preparing itself to attach in just a few seconds after climbing on to a human host (left). *Hyalomma lusitanicum* (right) prefers to avoid this host although it does sometimes prey on humans.

It has been reported that *H. marginatum* is very aggressive [66] and has a high affinity for humans [16]. In our experience, it has a higher affinity for humans and is more aggressive than *H. lusitanicum*. *H. marginatum* adults try to attach via the hypostome very quickly. This behavior was more evident when sampling was performed under conditions of high temperature/low humidity (40 °C, 25.5% relative humidity (RH)) than under lower temperatures (<20 °C) and higher humidity. Similarly, Estrada-Pena & Venzal (2007) [15] concluded that more ticks can be found at high temperatures and low relative humidity. However, *H. lusitanicum* adults spend more time searching for the best place to feed but generally prefer to avoid humans (Figure 2, Video S1). Nevertheless, it does sometimes attach to humans as well. Some authors suggest that this interspecific difference may be due to variations in the ability to identify suitable host signals owing to sensory system development [67]. These observations support Uspensky [68] who claimed that high temperature and low RH “stimulates ticks to use blood-feeding as the source of rehydration” as is the case with some blood-feeding mosquitoes [69].

### 3.2. Life Cycle

Natural life cycle duration depends on climatic conditions and host abundance but an estimation is possible by analyzing data from laboratory colonies. The immature stage life appears to be longer for *H. lusitanicum* (21–36 days, even where the cuticle hardening period is not taken into account) than for *H. marginatum* (14–26 days); the molt to adult period is similar (around 15 days) and adult feeding until gravid females depends on the presence of both sexes on the host and could be as short as 5 days for *H. lusitanicum* and 14 days for *H. marginatum* and up to 30 days for both species (Table 3). The entire cycle can be completed in 101-196 days for *H. lusitanicum* [37,70,71] and 90–167 days for *H. marginatum* [45,71,72]. Maximal egg production correlates closely with the size of the female but can consist of 14,500 and 15,500 eggs, respectively (Table 3). Thus, apart from the fact that one is three-host and the other a two-host tick, the rest of their biological cycle is similar.

Both species overwinter as unfed adults hidden in the field. In the case of *H. lusitanicum* two other ways were reported: males are also able to overwinter on hosts and engorged females may do it hidden in the field [35,36]. Ouhelli and Pandey [70] reported the ability of engorged *H. lusitanicum* females to delay egg laying when incubated at temperatures below 16 °C in the laboratory. Egg laying started when temperatures reached 25 °C and RH 84%. In our experience, field engorged females of *H. lusitanicum* detach in late autumn and winter, remain hidden and delay oviposition for three to five months, regardless of whether they are maintained at suitable conditions in the laboratory, suggesting a real diapause. Even in in vitro feeding assays, pre-oviposition is conditioned by the season during which adults were collected in the field [38]. *H. marginatum* engorged nymphs under unfavorable conditions suffer diapause delaying molting and overwinter for several months although suffering high mortality rates during this period [11].

The number of annual generations is influenced by the strain of the species, for example, *Hyalomma anatolicum*, a two-host life cycle tick, can have 3 to 4.8 annual generations [76]. Nevertheless, environmental conditions play a key role (climate, season, host availability, among other factors). Other *Hyalomma* species also have long life cycles [76,77,78]. Although it is possible to find engorged *H. lusitanicum* females throughout the entire year [35], *H. lusitanicum* and *H. marginatum* really only produce one generation per year.

**Figure 3:**
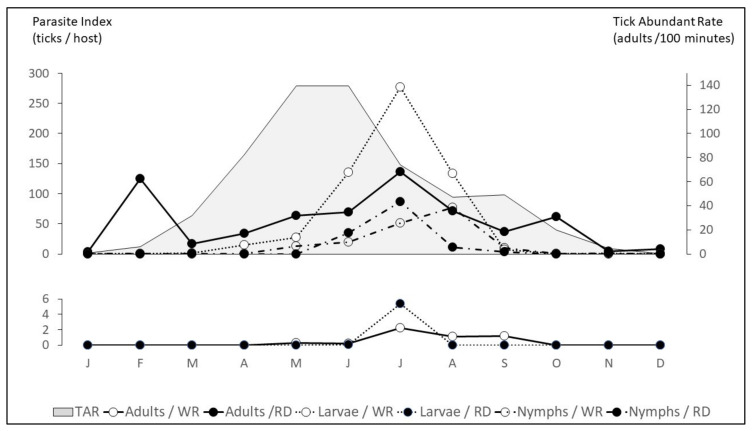
Seasonal pattern of main host infestation by *Hyalomma lusitanicum* and Tick Abundance Rate (host seeking adults) in a meso-Mediterranean area [32,35,36]. RD = red deer, WR = wild rabbit.

The pattern of host infestation of *H. lusitanicum* in the Iberian Peninsula has recently been described (Figure 3), and a description of *H. marginatum* life cycle under laboratory conditions by Yukari et al. [74]. It is possible to infer some differences. Immature stages of both species have a long activity period (Table 4) but *H. lusitanicum* is more active in spring to early summer while *H. marginatum* young are more active in the summer and autumn. In the 20th century *H. lusitanicum* reports indicate activity in the Iberian Peninsula from April to November [21,79] while *H. marginatum* is active all year round with maximum activity in spring [79]. In our experience in meso-Mediterranean areas, today *H. lusitanicum* adults remain active all year round [35] (Table 4) with a period of maximum activity from May to July and minimum activity in December and January, when only males are detected on red deer. After feeding, gravid females remain on the ground and the larvae parasitize wild rabbits between April and September with maximum activity in July. After molting, nymphs feed on rabbits or red deer and follow a similar pattern than larvae, from May to September with maximum activity in July and August [30,32,35,46,63]. Lastly, these nymphs develop into adults and the overwintering period varies depending on whether they can access a host and feed (last feeding recorded in October) or remain on the ground as non-fed adults. Engorged females drop off and remain in a morphogenetic diapause until February and host seeking adults on the ground remain inactive protected by leaf litter and react when conditions improve.

Host seeking *H. lusitanicum* adults can be collected during all the months of the year, with a high and long peak of activity from February to May–July (1.5 ticks/minute sampled), descending gradually until November [30,36] (Figure 3). In September-October, at the end of the theoretical questing season, there is a small peak coinciding with high host density related to breeding season. This could be the consequence of an increase in the questing activity of those adults with critical levels of lipid reserves [80,81]. Those males and females that cannot find a host during this last-chance period will be obliged to overwinter in the field and joined to males attached to red deer will produce the first engorged females in February. The offspring of these females will overlap with the offspring of overwintering engorged females.

In Africa, immature stages of *H. marginatum* feed on their first host between May and September and adults between March and November with a peak of infestation in April–May due to overwintering adults [30,53]. However, rising temperatures associated with climate change may modify this pattern for traditionally colder months [82,83]. In countries such as Iran, the highest levels of activity occur in August and September but in Pakistan activity follows a biannual pattern, between March and May and then from August to October [30,84,85].

Rising temperatures due to global climate change is a concern for the expansion of *H. lusitanicum* in new areas of Central and Northern Europe. Current weather conditions have prevented its establishment after an accidental import. However, if relative humidity decreases and temperatures rise above 15–20 °C (Table 5) for several weeks, the survival rate will increase significantly if enough hosts are available. In contrast, *H. marginatum* is able to support very cold temperatures and high humidity [86], so its likelihood of becoming established is higher.

### 3.3. Sex Ratio

The proportion of males/females on mammals provides very beneficial information on the reproductive aspects of population dynamics. In ixodids, this ratio can also be very useful insofar as it helps us to understand certain aspects of the life cycle, differences in survival rates, relationship to climatic and environmental conditions, estimations of the number of generations per year, fecundity, and so on. In this case, the monthly analysis of *H. lusitanicum*’s sex ratio, considered jointly with the feeding state of females, enables us to confirm that despite the length, at least four life cycles may start in the same year. This sex ratio remains constant during questing throughout the entire year (in a population of 6890 adults studied, mean = 0.99 ♂/♀, min = 0.50, max = 1.68 [36]). However, this ratio is more variable when infesting red deer (in a population of 8,978 adults studied, mean = 1.79 ♂/♀, min = 1.36, max = 2.63 [35]), especially in November and December when there are practically no females infesting the hosts in comparison to males. After mating with the last females in autumn, males remain on the host as long as possible waiting for the first females of the following year [35]. No information is available on the sex ratio of *H. marginatum* but it is assumed to be similar when infesting hosts given that males also remain attached whilst engorged females drop off after engorgement [11].

### 3.4. Questing/Host Seeking Strategies

Hard ticks are attracted by carbon dioxide, specific kairomones, substances excreted in the urine or by interdigital glands, and/or other specific signals (vibration, visual objects, ammonia or body heat) emitted by their usual hosts [88,89,90]. Questing is the behavior displayed by some ixodid ticks to access their host through the Haller’s organ on the first pair of legs. Ticks wait for long periods of time on vegetation. When they sense a host approaching, they stretch their front legs and hook on to their host’s hair coat [30]. Questing is highly influenced by the time of day in response to varying environmental conditions [91]. In questing species this behavior favors sample collection of larvae and nymphs by dragging, however this method is less efficient for adults and hunting species [30]. Many *Hyalomma* spp. prefer to actively seek their host using their well-developed sight, which allows them to visually recognize the host, and actively move towards it [89]. *Hyalomma asiaticum* can actually walk/run for a distance of up to 100 m [92].

**Figure 4:**
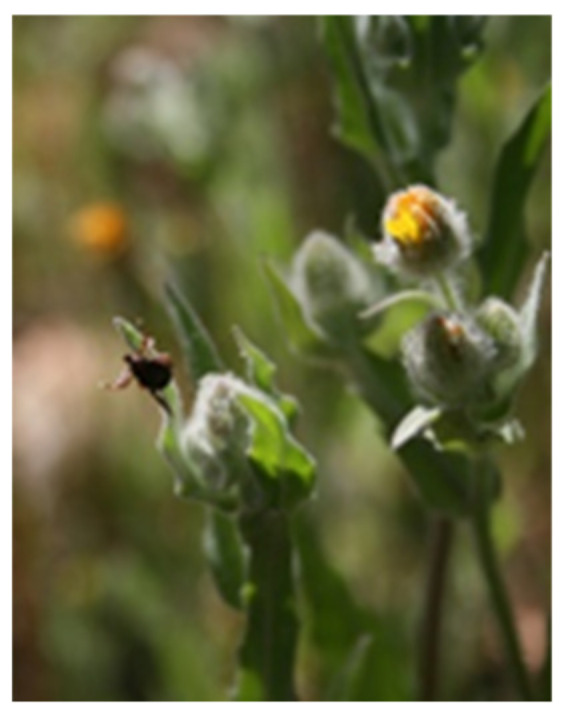
Questing *Hyalomma lusitanicum* with two front legs extended to expose the Haller organ to detect host stimuli.

*H. lusitanicum* adults are able to climb up on vegetation where they wait for a host with their two front legs extended to expose the Haller organ to detect host stimuli (Figure 4). However, for the most part they remain hidden on the ground or amongst vegetation cover and, when the host is detected, they use their sight and run quickly towards it (personal observations). Their eyesight also enables them to select the best hiding places [89] (Video S3). We concur with Strong [90] who reported that tick distribution in the field is not random. Indeed, there are a number of “hot tick spots” with the best environmental conditions (temperature, humidity, vapor pressure, season, wind, and plant height) where *H. lusitanicum* adults can easily be found (Video S4) [36,90]. Other species such as *Dermacentor variabilis* situate themselves along trails [88] but *H. lusitanicum* is mostly found in areas where the host remains for extended periods of time to rest or in the vicinity of feeders (personal observations). It is also possible to find adults of *H. marginatum* by dragging [62,93], but we do not believe that this is an appropriate sampling method, as reported by the European Food Safety Authority [94].

*H. lusitanicum* adults can be collected in winter by dragging only when temperatures are over 15 °C, but they truly start to regularly seek a host in March when, due to more favorable conditions, their numbers increase rapidly. The *H. lusitanicum* questing pattern is unknown in Central and Northern Europe. We found little data on the host seeking seasonality of *H. marginatum* in Europe, but in Africa there is a questing peak of overwintering adults in the spring, while immature stages are mainly active in summer, between May–June and September–October [30,53].

As a tick collection technique, dragging is not the most efficient method for hunting *Hyalomma* species, but it is a standard way to determine seasonal activity. The efficiency of this technique depends on the species of *Hyalomma* and methodological limitations. *H. marginatum* adults actively seek their hosts when they detect certain signals (vibration, visual objects, carbon dioxide, ammonia, and body heat) and can run towards a host for periods of ten minutes and over large distances [11,43]. This hunting behavior could be the reason for the low success rate when dragging, even when sampling is performed in the same pastures and during the same days when livestock is heavily infested (personal observations). *H. marginatum* is active in wet soil [11,48] and can survive short periods of submersion (5–7 days) [86] so, it is possible to find adults by dragging, even immediately after rainfall, when the soaked flag technique is useless. By contrast, host seeking adults of *H. lusitanicum* prefer dry environments and therefore the dragging technique is more successful if the flag remains dry. It is important to consider this aspect when sampling with a view to compiling field data able to support the design of control strategies against non-parasitic stages.

As mentioned, *H. lusitanicum* adults remain hidden and inactive until they are reactivated by compounds emitted in nature (handlers’ breath, among others) or artificial stimuli (dry ice). During Mediterranean summers when the weather is extremely hot and dry, *H. lusitanicum* adults remain inactive most of the day under the shade of trees and become very active at night (personal observation) (Video S2). In contrast, *H. marginatum* adults do not respond immediately to breathing compounds but once activated run fast towards the host (personal observations). There are no reports about nocturnal activity of *H. marginatum*.

### 3.5. Relationships to Climatic Parameters

Like most ixodids, *Hyalomma* spp. are particularly sensitive to environmental parameters (Table 5). *H. lusitanicum* is mainly distributed throughout the hot-summer Mediterranean climate (Csa) [35] whilst *H. marginatum* prefers hot desert climates (BWh) over hot-summer or warm-summer Mediterranean climates (Csa and Csb), cold desert climates (BWk) or cold semi-arid climates (BSk) [49].

Host seeking *H. lusitanicum* adults are more active on partially sunny days when the soil is dry, the grass is high, there is no wind, relative humidity is 10% to 60%, and the temperature is between 20 and 35 °C. However, activity is really low when temperatures fall below 10 °C or rise above 40 °C [34,36]. We do not have survival rate data for the immature stages of *H. lusitanicum* in the field but unfed larvae can survive for over eight months under laboratory conditions (25 °C and 85% RH) with natural light–dark cycle (Unpublished data). By contrast, *H. marginatum* prefers areas with higher humidity because its populations are regulated by rainfall and evapo-transpiration [95]. Rainfall is the main limiting factor in *H. marginatum* distribution that prefers high relative humidity (75‒100%) [11] and established populations are only maintained when the yearly temperatures accumulation is between 3‒4 °C and water vapor deficit is below an average of 15 hPa [53,96]. Low temperatures do not appear to affect them. Larvae are more active between 14‒16 °C and adults at 22–27 °C [53], although viable adults have been recorded at temperatures below −20 °C in Russia [86]. When the air temperature rises above 30 °C and the ground temperature above 45 °C, ticks prefer to hide or even burrow into the ground. Warmer autumns enable the molting of nymphs to adults thus decreasing the mortality rate and resulting in their gradual expansion [16].

### 3.6. Habitats and Geographical Distribution

*H. lusitanicum* is distributed throughout Southern Europe, Middle Eastern parts of the Mediterranean Basin, and Northern Africa [30,97], and *H. marginatum* in North Africa, Asia and Southern, and Eastern Europe [30,98] (Table 6). *H. lusitanicum* is especially easily found in the Iberian Peninsula where it is the most abundant *Hyalomma* species, due to suitable conditions enabling it to complete its life-cycle. It is adapted to the biotic and abiotic factors of different local meso-Mediterranean ecosystems, in particular oak and cork oak forests, even with some introduced species such as eucalyptus [36]. This type of forest offers a suitable refuge for hosts and ticks, enabling the latter to survive [99]. *H. marginatum* can also survive in a wide range of conditions and a variety of habitats including open arid habitats, marsh, steppe, savannah, and hill and valley scrubland biotypes [86]. However it is absent from contemporary and former European deciduous and mixed forest biotypes [11] where it has been replaced by other tick species of the genus *Ixodes, Dermacentor* and *Haemaphysalis* [86].

Both species are expanding to new areas and habitats, some even shared by the two of them [53,103]. During the 1990s, the distribution of *Hyalomma* ticks in the Iberian Peninsula seemed to be limited by the central mountain chain [79]. Today, however, both species have been reported in new areas. Moreover, *Hyalomma* ticks are not only expanding in their natural habitats but are also gradually extending into new environments, such as Central Europe [12,13]. There is also disturbing evidence of their adaption to new environmental niches [104]. It is therefore important to confirm whether the populations appearing in those new areas have become established.

Many factors could contribute to the spread of ticks: climate change, reforestation owing to the conversion of agricultural and grazing land into hunting areas or natural reserves, and increased animal and human movement, among others. The introduction of *Hyalomma* species into new areas is probably due to the presence of infested hosts resulting from human activity, movement of farm and wild mammals and migrating birds. The spread of *H. marginatum* by migratory birds is well documented [8,101,105]. In Zhemchuzhnyi Island (Caspian Sea) thousands of birds rest during their autumn migration south, and many of them are infested by engorged nymphs that overwinter and molt to adults the following spring [86]. However, although *H. lusitanicum* can be found on birds, the use of these hosts as an expansion mechanism does not appear to be very prevalent [85,100,106]. We agree with that premise, at least in the Iberian Peninsula, given that this species is clearly dependent on the rabbit population [32]. Furthermore, the period of maximum activity of larvae and nymphs (Table 4) takes place when most of the migratory birds have already flown North in late winter and early spring. Similarly, in their migration to the South, birds rest in the Iberian Peninsula when *H. lusitanicum* larvae are inactive. Changes in global weather are also responsible for the settlement of newly introduced populations, whereas previous weather conditions did not allow them to overwinter. Although there is an alleged negative correlation between *Hyalomma* abundance and altitude [49], they are also expanding vertically; both species can be found from 0 m to 1000 m above sea level in Iberian Peninsula (personal observations) and *H. marginatum* has been collected by flagging at 2000 m above sea level in Turkey [107], while in the past they were rarely found at high altitudes [31,87].

## 4. Conclusions

Although both species, *Hyalomma lusitanicum* and *Hyalomma marginatum,* are considered Mediterranean ticks, there are many differences that may affect distribution, abundance, and vectorial capacity in traditional and new habitats. For *H. lusitanicum*, a tick adapted to hot Mediterranean summers, global warming, reforestation, and wild fauna protection increase its distribution and abundance in its current habitats. In the case of *H. marginatum,* flourishing in temperate and more humid habitats, global warming has made Central Europe a new habitat for this species, introduced by migratory birds. It is important to set up a monitoring network using new tools, specifically developed for these species, to establish effective control measures. To that end, 21st century technological advances should be combined with traditional early 20th century tick ecology studies.

## Figures and Tables

**Table 1:** Known vectorial data of *Hyalomma lusitanicum* and *Hyalomma marginatum*.

	*Hyalomma lusitanicum*	*Hyalomma marginatum*
Vector demonstrated of	Coxiella burnetii (Q fever) Theileria equi Theileria annulata (Mediterranean theileriosis)	Babesia caballi (babesiosis) Coxiella burnetii Crimean-Congo Haemorrhagic Fever virus Rickettsia conorii (botonouse fever) Theileria annulata
Presence reported but transmission should be determined:	Anaplasma phagocytophilum Anaplasma platys Borrelia burgdorferi (Lyme disease) Crimean-Congo Haemorrhagic Fever virus Francisella tularensis (Tularemia) Rickettsia aeschlimannii Babesia caballi	Anaplasma phagocytophilum Anaplasma platys Babesia caballi Bahig virus Dhori virus Matruh virus Rickettsia aeschlimannii Theileria equi
Transmission ways	Transovarial Transstadial	Co-feeding Intrastadial Transovarial Transstadial Venereal

References: [17,19,20,21,22,23,24,25,26,27,28]

**Table 2:** General characteristics of the life cycle, main hosts and feeding habits of *Hyalomma lusitanicum* and *Hyalomma marginatum.* * Personal observations.

Characteristics	*Hyalomma lusitanicum*	*Hyalomma marginatum*
Behavior	Three-host tick Ditropic/telotropic * Immature ticks are endophilic/exophilic * Adults are exophilic One generation per year	Two-host tick Ditropic/monotropic Immature ticks are endophilic/exophilic Adults are endophilic/exophilic One generation per year
Host for immature stages		
Main hosts	Wild rabbits, hares	Wild rabbits, hares, passerine birds (blackbird, thrush bird, great tit, common accentor, red tailed bird)
Secondary hosts		
Larvae	Cattle, red deer, domestic dog, red fox, European polecat, rodents, garden dormouse, house rat, least weasel, red partridge and passerine birds	Rodents, hedgehog, passerine (larks and corvids) and galliform birds.
Nymph	Red deer, domestic dog, red fox, hares, house rat, least weasel, hedgehogs, bustard, red partridge, passerine bird	
Host for adults		
Main hosts	Red deer, cattle, camel, cattle, fallow deer, roe deer, sheep, wild and domestic goat, horse, domestic pig, wild boar	Camels, cattle, deer, goats, horses, sheep, wild boar
Secondary hosts	Leporids, wild rabbit, eagle owl, fox, hedgehog, mongoose, dog, human, bustard, ostrich, nocturnal birds of prey, gallifom and passerine birds	Donkey, Spur-thighed tortoise, weasel, fox, birds, human
Feeding sites	Immature specimens and adults on whole body More evident in areas with little hair: In small mammals on ears, belly or around eyes. In ungulates on hind quarters, inner side of the thigh, anus, udders * Congregations are very rare*	Immature specimens on birds and small-medium-sized mammals: head (mainly in and around the ears), face, neck and around the eyes Adults on ungulates only on hind quarters (mainly udders, scrotum, inguinal area and perineum). Typically appears in congregations

References: [20,32,35,45,46,47,48,49,50,51,52].

**Table 3:** Length (days) of the different phases of the life cycle of *Hyalomma lusitanicum* and *Hyalomma marginatum*. Numbers indicate the range of days (minimum or maximum) reported in the literature in studies performed in both laboratory and field conditions; in experimental infestation on animals or in vitro feeding assays. * Personal observations. ** Estimated length suggested by authors in the literature.

Life Cycle Phases	*H. Lusitanicum*	*H. marginatum*
Pre-oviposition	8–47	3–28
Oviposition	15–26	8–24
Hatching	22–40	20–40
Oviposition + hatching	34	
Larvae cuticle hardening **	8 *	5
Larvae feeding	4–8	26
Molt to nymph	12–16	15–30
Nymph feeding	5–13	
Larvae feeding+ molt to nymph + nymph feeding		12–26
Nymph cuticle hardening **		8
Molt to adult	11–22	15–30
Resting period of immatures **	21 *	21
Adult cuticle hardening **	30	8
Adult feeding	5–30	7–30
Whole cycle	101–196	90–167
Eggs laid per engorged female	629–14,519	2662–15,500

References: [11,37,38,45,51,70,71,72,73,74,75]

**Table 4:** Seasonal pattern of *Hyalomma lusitanicum* and *Hyalomma marginatum*. * Estimated by dragging.

*Hyalomma lusitanicum*	*Hyalomma marginatum*
Adult Host Seeking *	Complete annual pattern is unknown
Start to quest in March Main peak in May–June Later decline progressively Small increase in September–October	In Africa, peak of questing adults in April–May
Host infestation:	
Larvae: April-September (peak in May–June) Nymphs: May-November (peak in June–July) Adults: April to October (peaks in May–June and September–October)	Larvae-nymphs: May-June to September–October Adults: March to November (peak from April to June)

References: [21,30,32,35,36,59,79].

**Table 5:** Environmental preferences for field activity of *Hyalomma lusitanicum* and *Hyalomma marginatum*.

Parameter	*Hyalomma lusitanicum*	*Hyalomma marginatum*
Overwintering	unfed adults hidden in the field males on host engorged females hidden in the field	unfed adults hidden in the field engorged nymphs (high mortality)
Climate	Mediterranean steppe meso-Mediterranean	Mediterranean climate of North Africa and southern Europe Steppe, savannah and scrubland hill and valley biotypes
Preferences	partly sunny days dry soil no wind days tall grass or shrubbery	populations are regulated by rainfall and evapo-transpiration in summer (in Mediterranean basin) no strong wind
Best range of temperature	larvae/nymphs unknown adults: 20–35 °C	populations are regulated by the minimum temperatures in late autumn (in Eastern Europe and the Caucasus) larvae 14‒16 °C engorged nymphs tolerate 7–42 °C adults 22‒27 °C
Best range of Relative Humidity	adults 10–60%	engorged nymphs tolerate 0–100% adults 75‒100% although they may survive in low to moderate levels of humidity and long dry season during the summer months (but no survival in desert conditions)

References: [11,16,30,32,34,35,36,49,86,87].

**Table 6:** Habitats and distribution of *Hyalomma lusitanicum* and *Hyalomma marginatum.*

Factor	*Hyalomma lusitanicum*	*Hyalomma marginatum*
Habitat	Mediterranean forests Woodlands Scrublands	Arid open habitats, marsh, steppe, savannah and scrubland hill and valley biotypes They are absent from contemporary and former European deciduous and mixed forest biotypes
Geographical distribution	Europe (France, Italy, Portugal and Spain, including Canary Islands) North Africa (Algeria and Morocco) Occasionally in the United Kingdom	North Africa and Asia (Algeria, Armenia, Azerbaijan, Egypt, Ethiopia, Georgia, Iran, Iraq, Israel, Morocco, Sudan, Syria, Tunisia and Turkey) Europe (Albania, Bosnia and Herzegovina, Bulgaria, Croatia, Cyprus, France, Greece, Italy, Kosovo, the former Yugoslav Republic of Macedonia, Moldova, Montenegro, Portugal, Romania, Russia, Serbia, Spain and Ukraine) Occasionally in Germany, Hungary, Finland, Netherlands and the United Kingdom
Risk factors of spreading	Human and animal movement Reforestation of livestock farms	Human and animal movement Migratory birds (short distances) Degradation of agricultural lands especially after cattle pasture

References: [12,30,48,52,59,64,97,100,101,102].

## Supplementary Materials

The following are available online at https://www.mdpi.com/2075-4450/11/5/303/s1, Video S1. *H. lusitanicum* has not initial affinity to human, Video S2. *H. lusitanicum* has hunting activity during the night, Video S3. *Hyalomma lusitanicum* is hiding in the field, Video S4. Many *H. lusitanicum* adults are simultaneously stimulated by host presence.
