# Supplementary figures and images for: Comparative Ecology of Hyalomma lusitanicum and Hyalomma marginatum Koch, 1844 (Acarina: Ixodidae)
Source: Insects. 2020 May 13;11(5):303. doi: 10.3390/insects11050303 (PMC7290797; doi:10.3390/insects11050303)

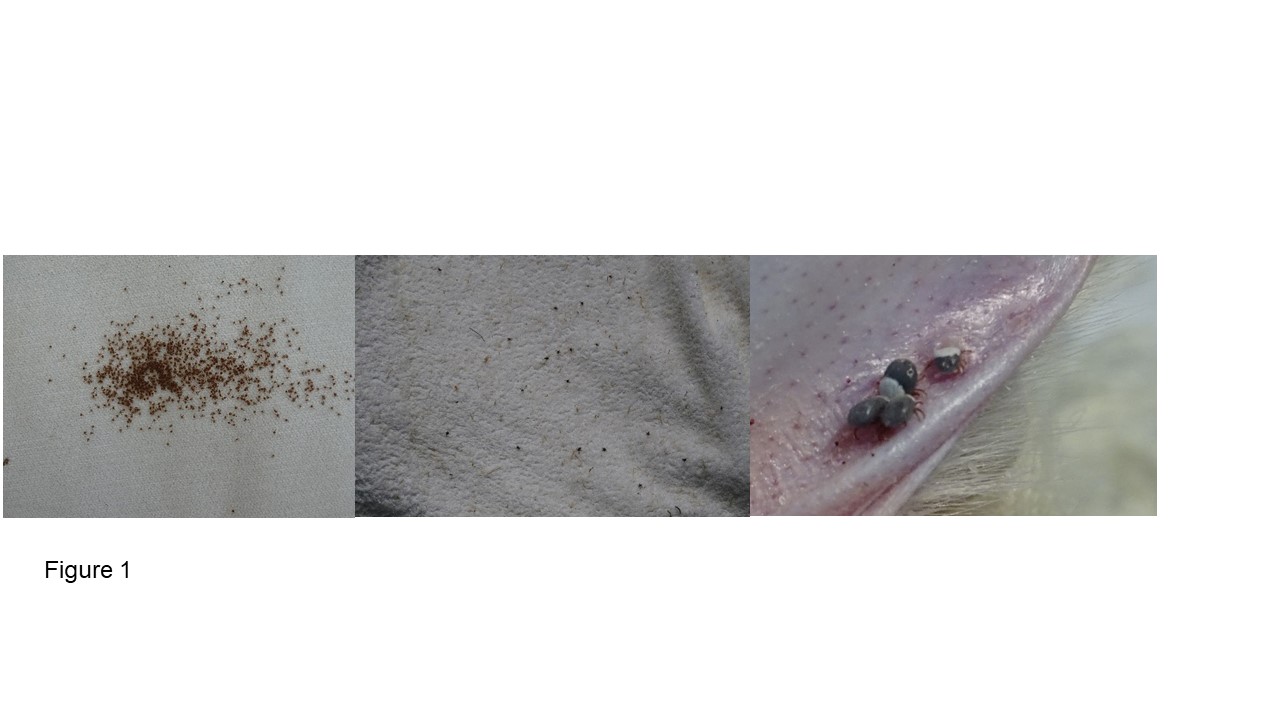

Supplement: Supplementary file 1 [file insects-11-00303-s001.zip › Figure 1.JPG]

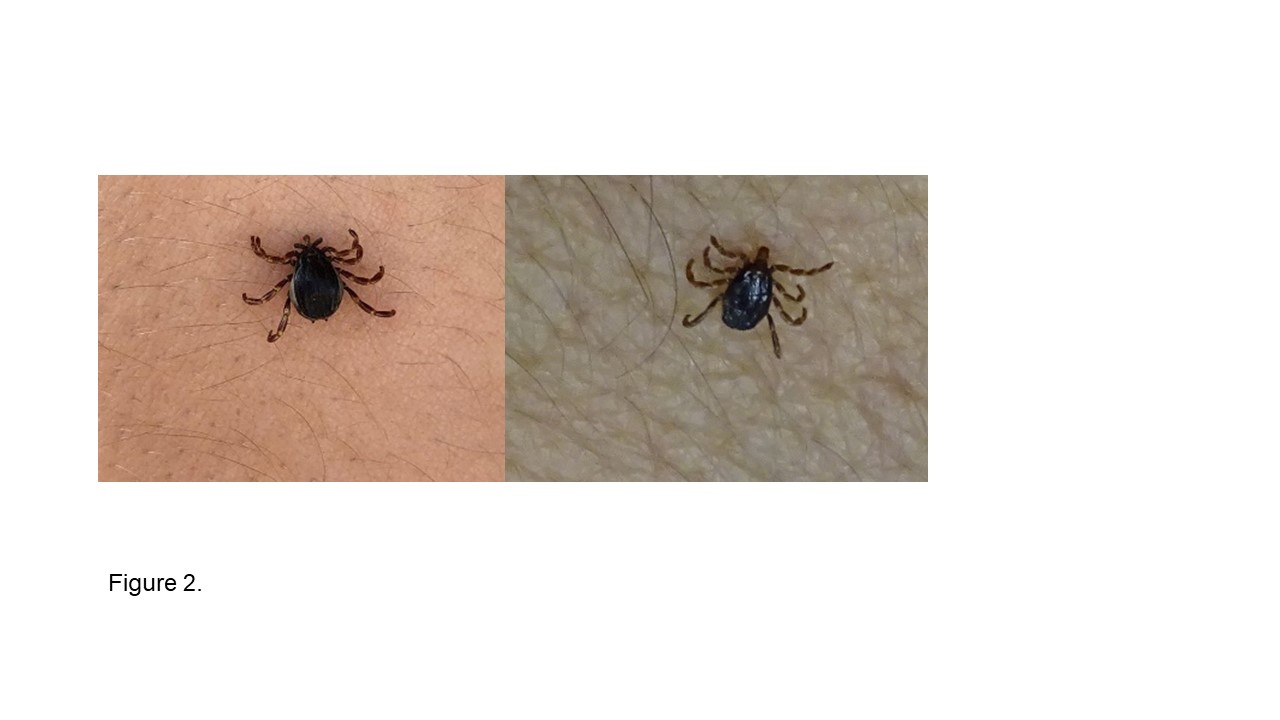

Supplement: Supplementary file 1 [file insects-11-00303-s001.zip › Figure 2.JPG]

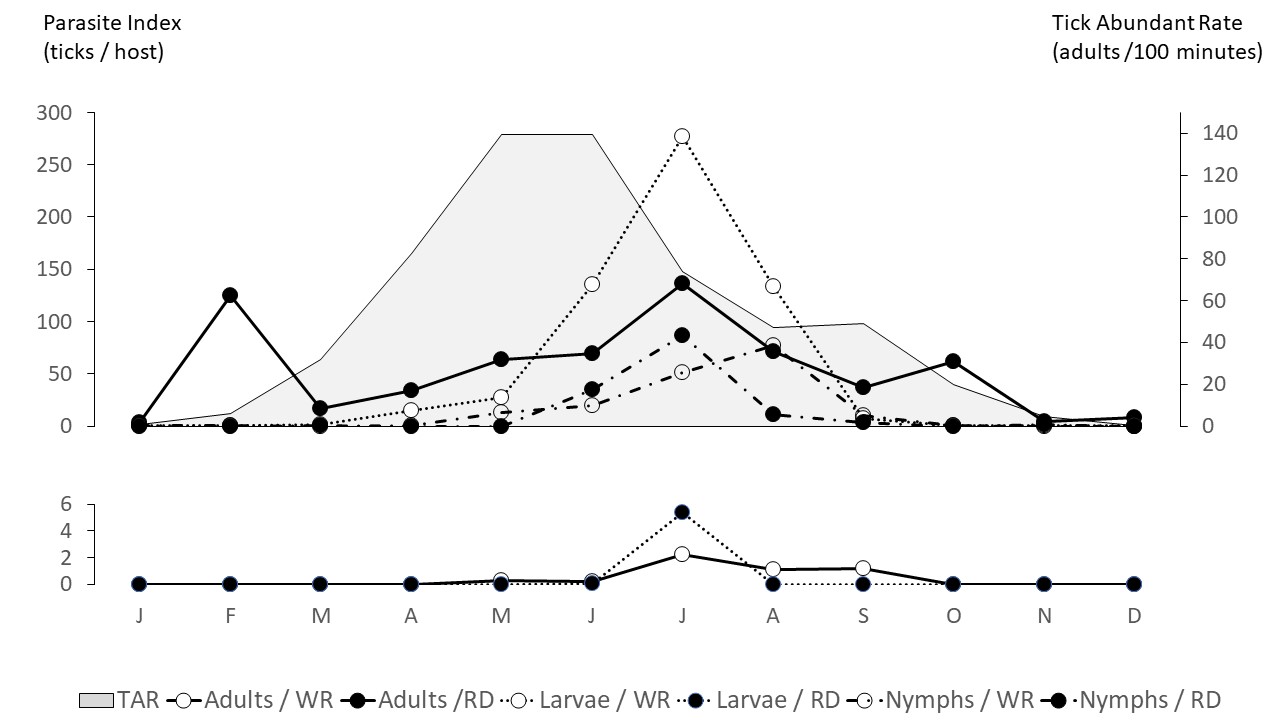

Supplement: Supplementary file 1 [file insects-11-00303-s001.zip › Figure 3.jpg]

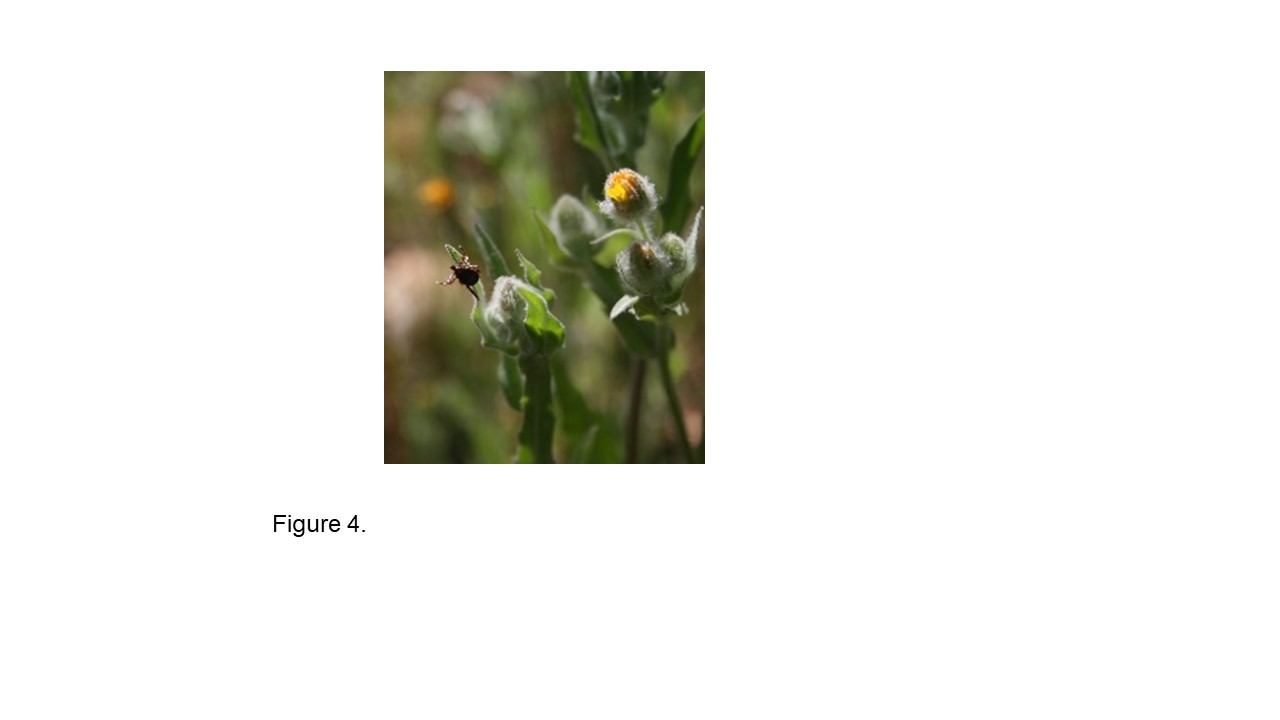

Supplement: Supplementary file 1 [file insects-11-00303-s001.zip › Figure 4.JPG]
